# Multiple expressions of “expert” abnormality gist in novices following perceptual learning

**DOI:** 10.1186/s41235-023-00462-5

**Published:** 2023-02-01

**Authors:** Gregory J. DiGirolamo, Megan DiDominica, Muhammad A. J. Qadri, Philip J. Kellman, Sally Krasne, Christine Massey, Max P. Rosen

**Affiliations:** 1 Department of Psychology, College of the Holy Cross, 1 College Street, Worcester, MA 01610 USA; 2 Department of Radiology, University of Massachusetts, Chan Medical School, Worcester, MA USA; 3 Department of Psychiatry, University of Massachusetts, Chan Medical School, Worcester, MA USA; 4 Department of Psychology, UCLA, Los Angeles, CA USA; 5 Department of Physiology, David Geffen School of Medicine, UCLA, Los Angeles, CA USA; 6 Department of Surgery, David Geffen School of Medicine, UCLA, Los Angeles, CA USA

**Keywords:** Gist, Perceptual learning, Histopathology, Abnormality, Detection

## Abstract

With a brief half-second presentation, a medical expert can determine at above chance levels whether a medical scan she sees is abnormal based on a first impression arising from an initial global image process, termed “gist.” The nature of gist processing is debated but this debate stems from results in medical experts who have years of perceptual experience. The aim of the present study was to determine if gist processing for medical images occurs in naïve (non-medically trained) participants who received a brief perceptual training and to tease apart the nature of that gist signal. We trained 20 naïve participants on a brief perceptual-adaptive training of histology images. After training, naïve observers were able to obtain abnormality detection and abnormality categorization above chance, from a brief 500 ms masked presentation of a histology image, hence showing “gist.” The global signal demonstrated in perceptually trained naïve participants demonstrated multiple dissociable components, with some of these components relating to how rapidly naïve participants learned a normal template during perceptual learning. We suggest that multiple gist signals are present when experts view medical images derived from the tens of thousands of images that they are exposed to throughout their training and careers. We also suggest that a directed learning of a normal template may produce better abnormality detection and identification in radiologists and pathologists.

## Introduction

Improving perceptual expertise in medical image interpretation is critical for patient care. The first step in diagnosis often is the visual categorization of a potentially significant clinical finding in a medical image (i.e., normal or abnormal). All future medical steps are dependent on the efficacy and accuracy of this original visual categorization. The human visual system has been shown to be very good at categorization with the ability to extract some category information about an object or a scene even with very limited exposures, as short as 100 ms (Potter, 1976). Moreover, participants can differentiate between two categories at shorter durations (Thorpe et al., 1996), with evidence for some abilities of categorization at even shorter intervals, as low as 20 ms (Greene & Oliva, 2009a, 2009b). Greene and Oliva (2009b) argued that rapid categorization is dependent in part on global properties. Within this global framework, the initial visual representation constructed by the visual system is at the level of the whole and not parts (Oliva & Torralba, 2006). Instead of local geometric and part-based processing (Biederman, 1987), this framework posits that global properties reflecting structure, layout and function could act as primitives for categorization.

This rapid visual categorization at low exposure durations has also been seen in medical experts for medical images (Brunye et al., 2021; Evans et al., 2013, 2016, 2019; Kundel & Nodine, 1975; Kundel et al., 2007). In a seminal study (Kundel & Nodine, 1975), a 200 ms presentation of a chest radiograph led to an abnormality categorization accuracy of 70%. Furthermore, with brief 250 ms or 500 ms presentations of a mammogram, radiologists can discriminate between normal and abnormal at significantly greater than chance levels (Evans et al., 2013, 2016, 2019). The visual categorization of normal or abnormal in medical images after a brief visual display may itself rely on two distinct visual processes including a critical primary global signal (Evans et al., 2013, 2016, 2019; Kundel et al., 2007).

First proposed by Kundel and Nodine (1975; see also, Kundel et al., 2007), experts obtain a global impression of the image which constrains their subsequent search locations for finding the abnormality. In this model, the first global process is a holistic comparison process which determines if the current medical scan deviates from an internal representation of a normal scan (Kundel & Nodine, 1975). This information is then used in a second process to guide attention and the eyes to the locations in the scan which deviated from that normal template (Kundel et al., 2008). Hence, the global signal contains location information of the abnormality (Kundel & Nodine, 1975; see also, Kundel et al., 2007, 2008).

Recently, this view of the nature of the global signal has been challenged (Evans et al., 2013, 2016, 2019). Rather than containing location information, Evans et al. (2013, 2016, 2019) and Gandomkar et al. (2021) have argued that the first process is a global abnormality signal devoid of location information. This first global signal (“gist”) simply provides information that something is abnormal based on a global implicit extraction of statistics across the whole image allowing for abnormality detection without containing any location information about where the abnormality lies or even why the image is being called abnormal. Some evidence for this global gist signal being solely a global implicit abnormality signal without localization information of the abnormality (c.f., Carrigan et al., 2018) is that with a brief (500 ms) glance at a mammogram, radiologists can detect gist abnormality in mammograms that do not contain a distinct lesion. And, even report abnormalities at significantly above chance levels for the mammogram of the healthy breast contralateral to the breast with a malignancy (Evans et al., 2016). Evans et al. (2013, 2016, 2019) and Gandomkar et al. (2021) suggest that an initial non-selective visual pathway which extracts global summary statistics about the image could implicitly alert the radiologist of a general “abnormality” signal, but a second stage of processing requiring focused attention is needed to localize any specific abnormality that may be present (see also, Wolfe et al., 2011).

While the exact perceptual features that support this “gist signal” remain unclear, it is hypothesized that the extensive visual expertise of the radiologist produces both this initial global “gist signal” and the subsequent localization of the abnormality (Carrigan et al., 2018; Drew et al., 2013a, 2013b). Specialized medical training and continuous high-volume reading of images give rise to a fine-tuning of the perceptual system of the radiologist. With increases in the number of slices for a CT or MRI scan, this fine-tuning of the visual system seems highly plausible given that the average radiologist must see and interpret one image every 3–4 s for 8 h straight (McDonald et al., 2015) to complete a typical workload day. Hence, radiologists have extensive visual experiences that leads to enhanced and finely tuned visual systems which gives rise to the ability to detect abnormalities (Drew et al., 2013b; Krupinski, 2010; Manning et al., 2006; Nodine et al., 1999; Rubin et al., 2015) including this primary gist signal. There is ample evidence of significantly better performance by radiologists than non-experts in medical visual search (Nodine et al., 1999; Quekel et al., 2001; Rubin & Krupinski, 2017; Rubin et al., 2015; Waite, 2019). For example, Kundel et al. (2008) demonstrated that radiologists go to the location of a breast abnormality within 1 s of viewing the mammogram in over 65% of trials.

The radiologist’s visual system is specialized for localization of abnormalities but not generalized visual search, as abnormality localization is better in experts than novices but general search tasks (i.e., find “Waldo”) are no different in medical experts than novices (Nodine & Krupinski, 1998). Hence, the highly trained, specialized, and practiced visual systems of radiologists can provide a global gist signal and rapidly localize the location of the abnormality. However, recent advances in perceptual training have demonstrated that extensive medical training and years of perceptual experiences with medical images is not necessary to localize an abnormality (Chen et al., 2017; Johnston et al., 2020; Kellman & Krasne, 2018; Krasne et al., 2013; Sowden et al., 2000; Xu et al., 2016).

Perceptual training refers to any training whose goal is to improve perceptual skills, such as the ability to recognize and categorize an image. The learning that results from this training is perceptual learning (Gibson, 1969; Kellman, 2002; Posner & Keele, 1968; Sowden et al., 2000), where instead of learning to interpret an image by following a set of explicit didactic rules, observers can instead be rapidly trained to visually determine a category (even in the absence of conscious knowledge). More than 50 years ago, Posner and Keele (1968) demonstrated that recognition of exemplars of a visual category could be categorized even in the absence of explicit knowledge of the category or its prototype.

Studies (Chen et al., 2017; Krasne et al., 2013; Sowden et al., 2000; Xu et al., 2016) have investigated perceptual training in the context of medical abnormalities and have demonstrated that abnormality localization can rapidly be improved with minimal perceptual training in novices. Sowden et al. (2000) trained novices to detect differences in X-rays and following 4 days of training on exemplars with classification feedback, accuracy improved by 10%. Chen et al. (2017) used 5 training blocks in a single day on categorizing hip fractures in X-rays and found that novices improved by ~ 20%. In a study by Xu et al. (2016), undergraduates were trained on 100 images of a melanoma and normal histology and were given feedback on their accuracy of categorization after each image. This training significantly improved their detection (*d*′) of skin abnormalities. When tested on a novel set of images, their false alarm rate also significantly decreased. Another study (Johnston et al., 2020) trained naïve participants on appendicitis and demonstrated that perceptual learning rapidly improved performance and generalized to a novel set of images with 80% accuracy. Moreover, the CT scans that novices found difficult to localize were the same images that expert radiologists also found difficult. Most relevant to our current study, Krasne et al. (2013) used a perceptual-adaptive training procedure for 1st and 2nd year medical students on skin histopathology images. Medical students received multiple trials based on adaptive perceptual learning (Mettler et al., 2016) with immediate feedback of their accuracy. Despite only ~ 17 min of visual training, mean categorization accuracy of skin histopathology increased by ~ 13%. Perceptual training can give rise to accurate localization on CT scans, X-rays, and histology images in naïve participants without medical training or years of experience with those specific visual medical images. Brief perceptual training improves diagnostic accuracy in novices, this improvement generalized to novel images, and the same scans that novices find challenging are the scans that experts find challenging. In short, after a brief perceptual training period, novices’ visual systems seem to be fine-tuning for abnormality localization in a similar way as experts.

Perceptual training applies across multiple domains of medical expertise (including skin histopathology) and seems to enhance the visual system of novices to perform accurate abnormality localization. What remains unknown is if novices after perceptual training demonstrate the initial global gist signal that experts show. In short, while perceptual training has been demonstrated to improve the 2nd stage of processing of localizing the abnormality that is seen in experts, what is unknown is if perceptual training also creates the ability to rapidly process a medical image and extract a global signal about abnormality (or a global signal about the location of an abnormality). In the following experiment, we directly tested this question.

The aims of the present study were to extend the previous research on perceptual learning in medical images to see if novices who have been perceptually trained on histology images would also show a global abnormality signal and to explore whether the global signal was about general abnormality or lesion information at a specific location. To determine this first aim we showed naïve participants who were perceptually trained on a brief skin histopathology module (Krasne et al., 2013), a 500 ms image of a histology image which was then masked to stop further perceptual processing (Breitmeyer, 1984) and asked them to determine if the histology image was normal or abnormal. This paradigm replicates that used in trained medical professionals to demonstrate “gist” (Evans et al., 2013, 2016, 2019; Gandomkar et al., 2021) and provides direct evidence for or against this signal arising in perceptually trained novices. Our second aim was to explore if the signal extracted by naïve participants provided only a general abnormality signal or gave rise to specific local information about the abnormality. Thus, after making an abnormality judgment, participants were asked to categorize the abnormality into one of four distinct skin histopathologies learned during perceptual training. In addition, we asked participants about how they made their decision (guess or know) to help determine if the signal was related to information consciously learned during perceptual training or implicit information (as suggested by Evans et al., 2013) gathered by the perceptual system during the perceptual training in which case participants would report “guessing” rather than “knowing.” Finally, we asked what information during perceptual training was important for the abnormality signal that perceptually trained naïve participants used. To be explicit, was abnormality detection related to how well participants perceptually learned the abnormal or, as Kundel and Nodine (1975) and Kundel et al. (2008) have suggested, related to the creation of a normal template?

## Methods

### Participants

Twenty undergraduates (6 males, 14 females; all aged between 18 and 21 years) took part in the experiment and received course credit or reimbursement for their participation. All reported normal or corrected-to-normal vision and normal color vision. All participants gave informed consent and were treated in accordance with the ethical standards of the Declaration of Helsinki and the American Psychological Association. All participants were naïve to skin histopathology and had not received any previous training prior to the start of the experiment.

### Materials and procedure

**Fig. 1:**
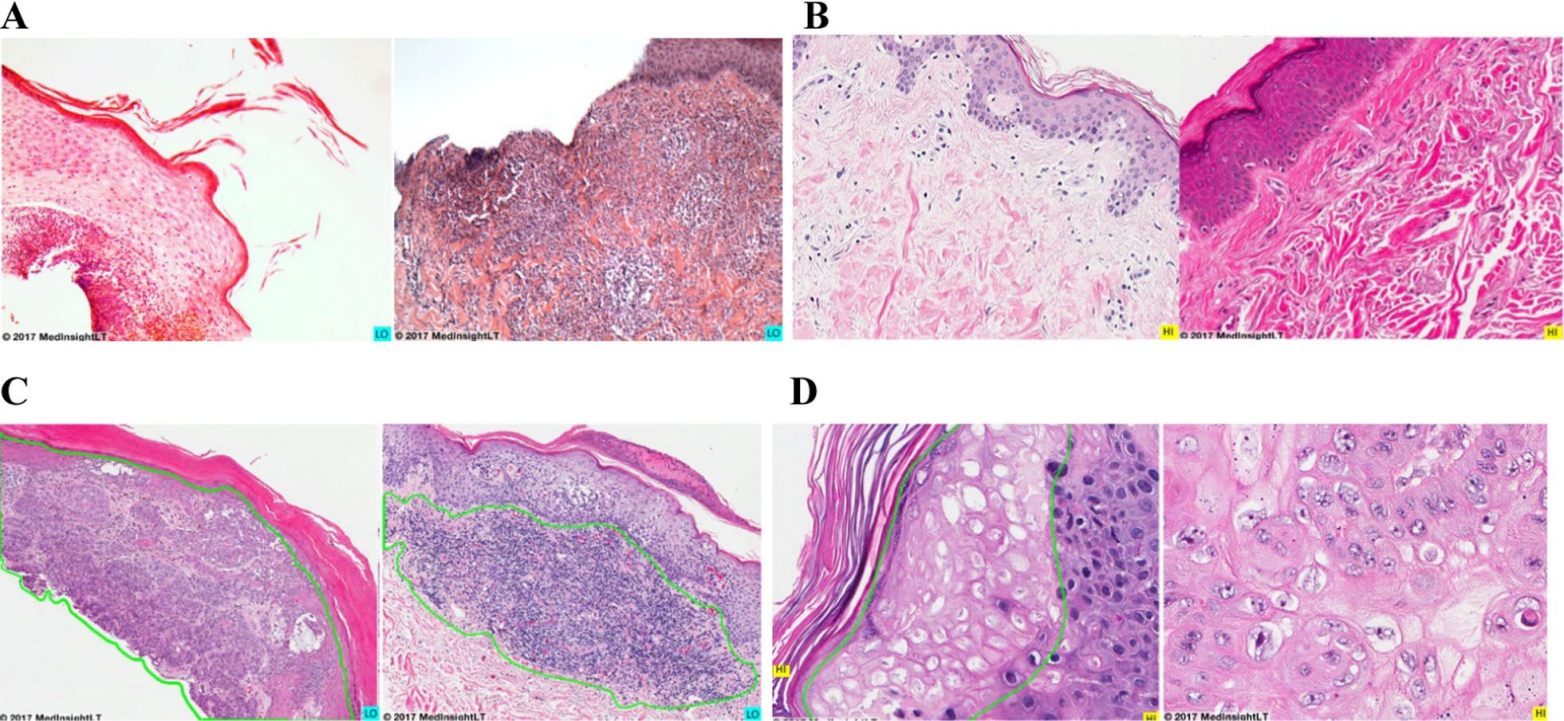
**A** Two examples of acute inflammation having very different global and local perceptual features. **B** Two examples of normal histology having very different global and local perceptual features. **C** The histopathology of an example of Neoplasia (left) and of chronic inflammation (right) having very similar local features (here highlighted by green borders which are only presented here for the reader and were not presented to participants) which are needed to identify the category. **D** The histopathology of an example of cell and tissue injury/repair (left) and of neoplasia (right) having very similar local regions (again highlighted by green borders which are only presented here for the reader and were not presented to participants) which are needed to identify the category

Stimuli were presented on a 21.5″ iMac placed at a viewing distance of ~ 65 cm from the participant. The resolution of the screen was 1920 × 1080 pixels. Exemplars from a pool of 298 color histology images of skin were presented at 462 × 361 pixels. Histology images (see Fig. 1) were divided into 5 categories (4 histopathology: acute inflammation, chronic inflammation, cell and tissue injury/repair, and neoplasia; and normal histology cell tissue). Naïve participants first completed a perceptual and adaptive learning module (Krasne et al., 2013) to teach them to identify normal skin cells and the 4 types of skin histopathology. Following perceptual training, participants immediately completed a 2nd phase of testing of abnormality detection and categorization following a brief presentation of a histology image.

### Histopathology perceptual and adaptive learning module (PALM)

**Fig. 2:**
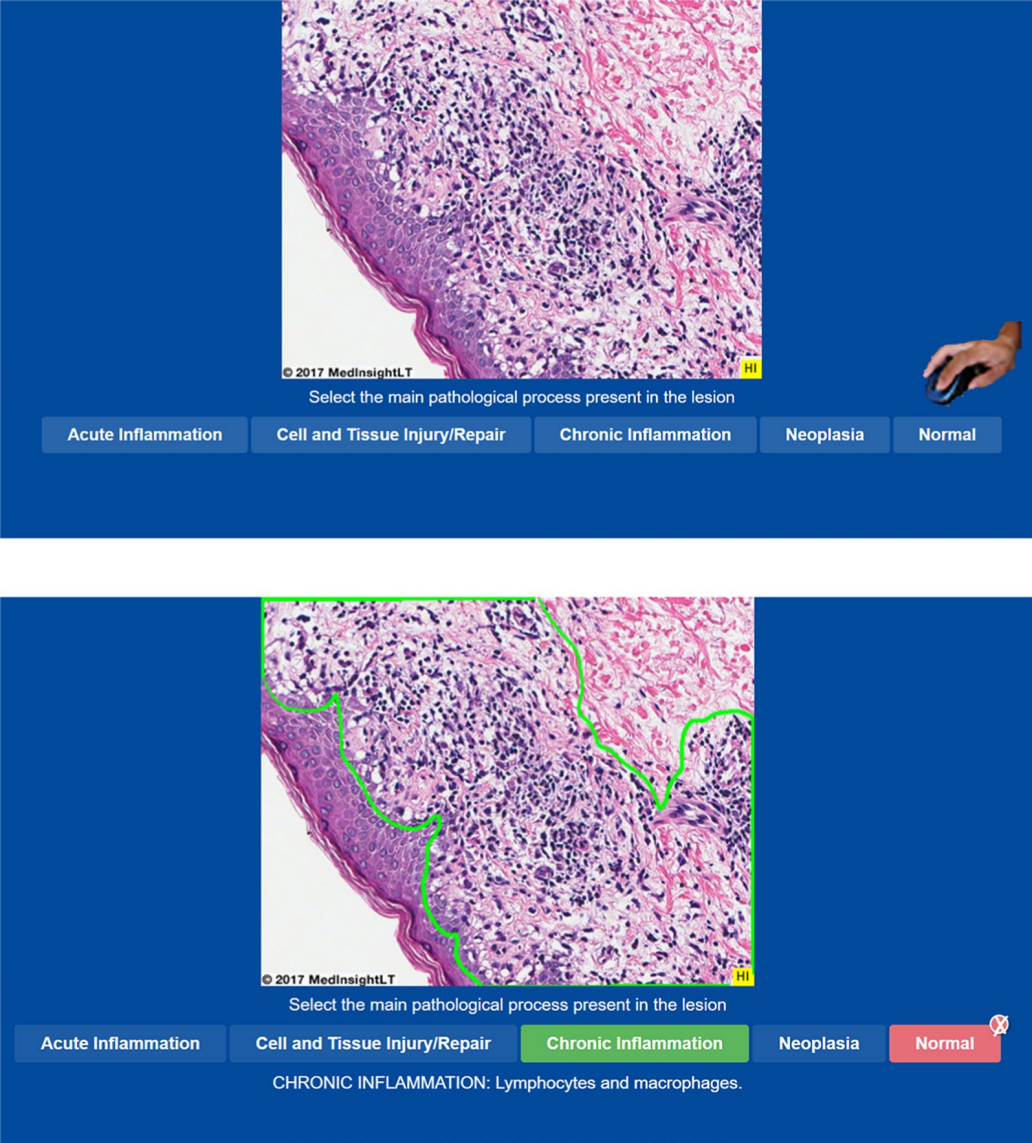
Schematic of perceptual learning. Participants saw a histology image and made a judgment of its category. They then received feedback on their answer. If the image was one of the four histopathology categories, the area of pathology was then highlighted

The PALM module (Krasne et al., 2013) combines perceptual learning with an adaptive learning system to accelerate pattern recognition skills and transfer (Kellman & Krasne, 2018; Mettler et al., 2016) to teach the different histology categories. For full details, see Krasne et al. (2013), but below we summarize the perceptual and adaptive learning. The adaptive sequencing adjusts priorities after each trial based on learner speed and accuracy, as well as the number of trials since the category was last presented. To achieve mastery of a category (and hence retire exemplars of that category), participants had to achieve three consecutive identifications correctly with each answer given within a specified response time (see, Krasne et al., 2013). Hence, the number of exemplar images from each histology category that participants saw during training differed based on the speed of acquisition of perceptual learning. Initial presentations of image categories were randomized, and then, presentations were subsequently sequenced according to individual priority scores for each learner, with the constraint that there were at least 3 intervening images between consecutive presentations of the same category. For any given training trial, a histology image was presented with each of the 5 categories (4 histopathology categories and normal) presented below the image (see Fig. 2). Once a category option was selected by a mouse click, participants were given feedback on the correct answer. If the image was a pathology, the area in the histology image showing the pathology was highlighted. After participants had retired every category, they then moved on to a brief assessment where they were randomly presented with 20 novel histology images and had to categorize each of the histology images. No feedback was given during the assessment. Following the assessment, participants then completed the gist evaluation.

### Abnormality detection and categorization task

Participants completed one block of 298 trials with 10 practice trials to start and then 144 normal and 144 abnormal histology images intermixed and randomized. Histopathology images were divided into the 4 categories (acute inflammation, chronic inflammation, cell tissue injury/repair, and neoplasia) with 36 exemplars from each category. The histology images used for testing were composed of both “familiar” histology images (those images seen during learning or assessment) and “novel” histology images (images not previously seen). The number of images that were novel during testing varied for each participant based on how many histology images it took for them to reach criterion during the perceptual learning.

**Fig. 3:**
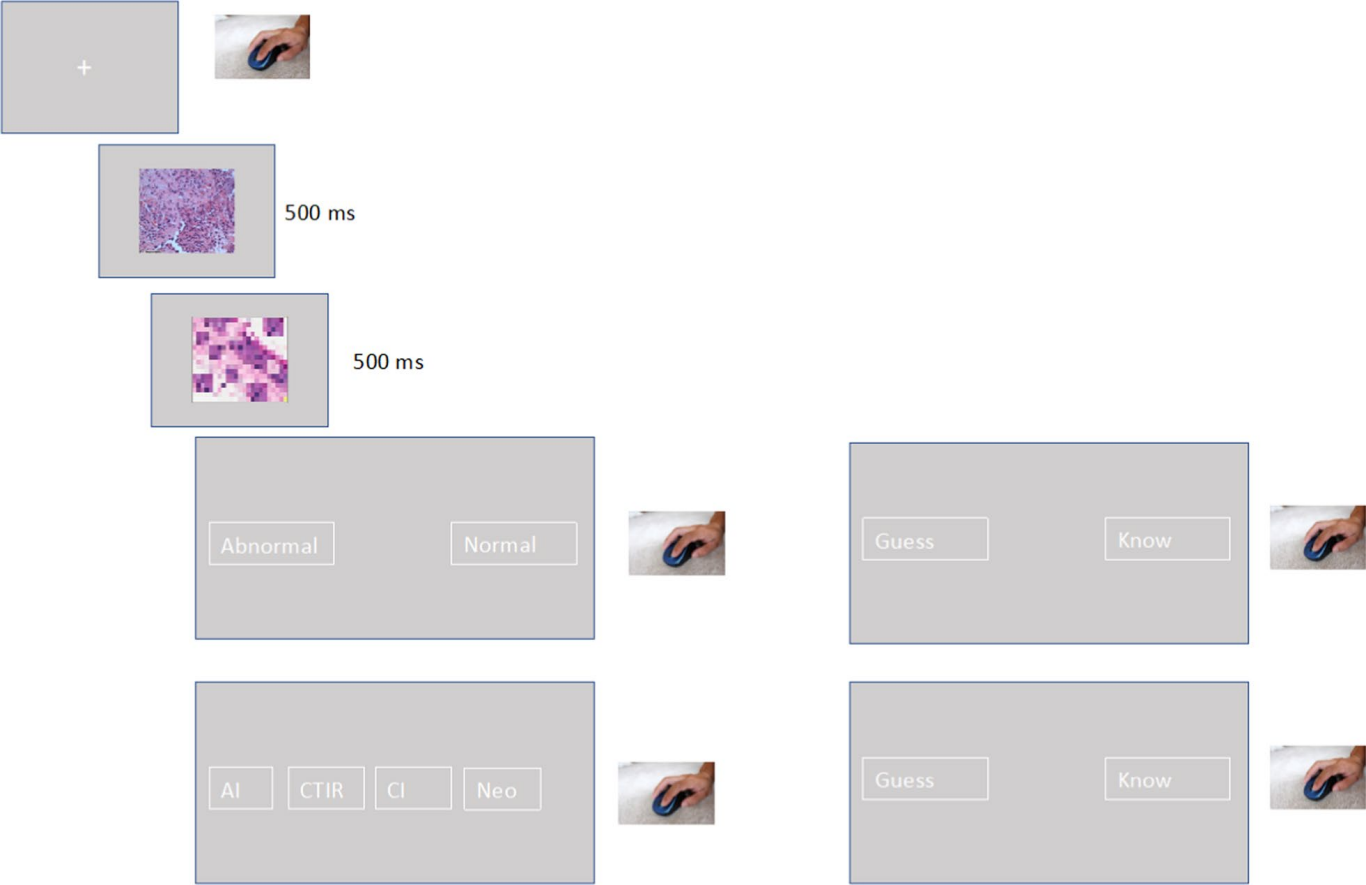
Schematic of gist experiment. Participants saw a brief (500 ms) flash of a histology image which was then masked. They made a normal/abnormal judgment and then reported how they made this judgment. If they had selected an abnormal judgment, after reporting how they made that judgment, they were then presented with the 4 histopathology categories and selected one of them. They then reported how they made this category decision

Each trial (see Fig. 3) began with a gray screen showing a central black fixation cross (Times New Roman, Bold 36 Font). The fixation was presented until participants clicked the mouse. Immediately after the mouse click, a histology image was presented for 500 ms followed by a mask for 500 ms. The mask was composed of parts of multiple histology images randomly intermixed and pixelated and was 462 × 361 pixels in size. After the offset of the mask, two boxes (each 248 × 86 pixels) were presented at the bottom of the screen with abnormal on the left and normal on the right (each box 420 pixels from their respective edge of screen). Participants had to make their response by moving the mouse to the box that contained their judgment and clicking on it. After responding, a new screen with two boxes containing guess or know were presented (same sizes and distance as previous boxes), and participants clicked on the appropriate box to indicate how they made their judgment. After indicating how they made their decision, if participants selected abnormal, participants were presented with each of the 4 histopathology categories in boxes (each 248 × 86 pixels) at the bottom of the screen and selected which histopathology category the abnormal image belonged to by mouse clicking on that box. After this category judgment, participants again indicated how they made their category decision by selecting the guess or know boxes on the next subsequent screen (see Fig. 3). There was a 500 ms inter-trial interval before the next fixation would appear.

### Analyses

For statistical analyses, we measured the accuracy for the abnormal and normal detection and used these measures to compute a *d*′ score for abnormality (Macmillan & Creelman, 1991). Using SPSS v.28, we compared the *d*′ prime scores against chance (0) using one-sample *t* tests, and paired *t* tests to compare differences in *d*′ abnormality detection when participants reported knowing and when they reported guessing; and when comparing familiar versus novel histopathology images.

This design explored not only abnormality detection during rapid presentation but also the subsequent categorization of these same histopathology images. Because multi-item categorization judgments can be influenced by bias similar to two-alternative procedures, we sought a bias-independent measure of performance akin to the signal detection analysis above. To this end, we employed category-specific *d*′ computations and averaged them together to obtain an overall categorization *d*′. In this analysis, for any given target category, the non-target category histology images were labeled as false alarms if the target category label was applied, and correct rejections if not applied. Correct responses on category target images were labeled as “hits,” and incorrect responses on category target images were labeled as “misses.” To ensure the stability of these analyses, only those categories with at least 10 observations within and across the category were considered (i.e., permitting gradations of 10% for hit rate and false alarm rate; this eliminated one participant’s data). Again, we compared the *d*′ scores for correct category identification against chance (0) using one-sample *t* tests, and paired *t* tests to compare differences in category identification when participants reported knowing and when they reported guessing.

In addition, starting with naïve participants and using a rigorous, computerized adaptive learning module allowed us to examine how participants’ abnormality detection and abnormality categorization reflected differences in perceptual learning. To this end, we conducted stepwise regression analyses starting from null models and adding predictors to improve the fit of the model. Each regression analysis used the observed learning data: (1) the average accuracy for abnormal histology images during training, (2) the average accuracy for normal histology images during training, (3) the total number of abnormal histology exemplars seen (including repetitions of the same histopathology image) before reaching criterion, (4) the total number of normal histology exemplars seen (including repetitions of the same histology image) before reaching criterion, (5) the total amount of time to complete the PALM, and (6) performance accuracy during assessment—to explain the participants’ performance on abnormality detection and abnormality categorization.

## Results

### PALM

On average, participants completed the PALM training in 45.4 min (SE = 4.57 min). Participants saw, on average, 258 (SE = 28) abnormal histology exemplars (including repetitions of the same images) until they reached criterion on all 4 histopathology categories. They also saw 57 (SE = 9) normal histology exemplars on average (including repetitions of the same images) until they reached criterion on normal histology. Participants achieved 40% accuracy (SE = 2%) for abnormal histopathology images and 42% accuracy (SE = 3%) for the normal histology images during their perceptual learning. After training, participants were significantly better (M = 42%, SE = 3%) than chance (20%) on their ability to classify novel histology images on the assessment, *t*(19) = 7.23, *p* < 0.001, *d* = *1.62*.

### Abnormality detection

**Fig. 4:**
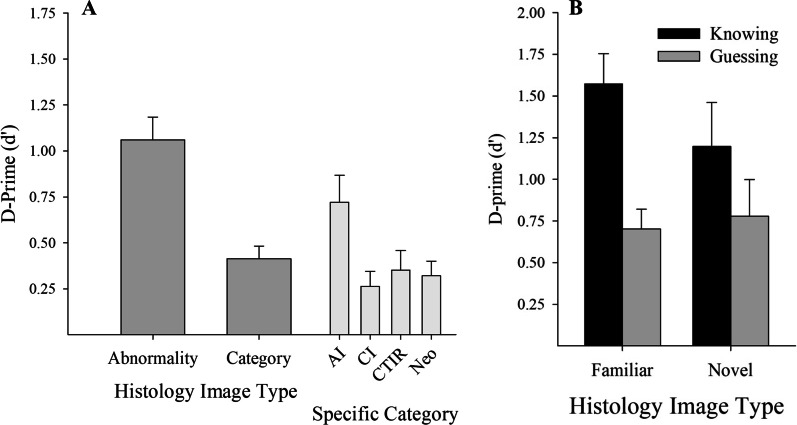
**a** Naïve participants’ ability following perceptual learning i abnormality detection (left) and in category identification (right). **b** Naïve participants’ abnormality detection ability with novel and familiar histology images, computed separately for self-reported guessing versus knowing

Participants demonstrated abnormality detection (mean *d*′ = 1.06, SE = 0.12), above the chance level of 0 (see Fig. 4a), with *t*(19) = 8.5, *p* < 0.001, *d* = 1.9, even with a brief 500-ms exposure of a histology image followed by a mask to stop further perceptual processing. Whether these responses and judgments resulted from perceptual category learning or from a memory-based process was determined by considering histology images previously seen (Familiar) versus novel histology images. For *Familiar* histology images that were seen during either learning or assessment (mean *number of exemplars N*_E_ = 222.8, SE = 3.9), participants showed significant abnormality detection, with *d*′ = 1.10, SE = 0.13. *Novel* histology images (*N*_E_ = 65.3, SE = 3.9) also showed significant abnormality detection rates (*d*′ = 1.03, SE = 0.19); in both cases, participants were above chance (*t*s(19) > 5.5, *p* < 0.001, *d*s > 1.2). Importantly, participants performed statistically equivalent BFat abnormality detection independent of familiarity, *t*(19) = 0.5, *p* = 0.604. Because this null result may suggest important similarities in the processing between familiar and novel histology images, we examined this in a Bayesian framework to identify the degree of support for the null hypothesis. This analysis showed substantial evidence (BF = 0.20, using diffuse prior) in support of the null hypothesis. Thus, during abnormality detection, participants were using an effective, generalizable process in determining abnormalities that applied to novel histology images as well as familiar histology images.

#### Self-reported process

As shown in Fig. 4b, even when participants reported using a guessing process (*M* = 45.2% of responses, SE = 5.6%), they remained above chance for abnormality detection (*d*′ = 0.68, SE = 0.13; *t*(19) = 5.3, *p* < 0.001, *d* = 1.2). When participants reported a knowing process, their abnormality detection was also significantly above chance (*d*′ = 1.44, SE = 0.19, *t*(19) = 7.6, *p* < 0.001, *d* = 1.7), and this performance was significantly better than when they reported guessing (*t*(19) = 3.8, *p* < 0.001, *d* = 0.9). Most telling, abnormality detection extended to novel items (see Fig. 4b), even when reporting guessing (*d'* = 0.78, SE = 0.21, *t*(19) = 3.6, *p* = 0.002, *d* = 0.80). A repeated measures ANOVA (familiarity × self-reported-process, dependent variable of *d'*) reported no main effect or interaction with familiarity (*F*s(1,18) < 1.7, *p*s > 0.204) and affirmed better performance when participants reported knowing than guessing (*F*(1,18) = 8.9, *p* = 0.008, *η*^2^_p_ = 0.33). These data reveal participants were able to differentiate between abnormality decisions where they had accurate knowledge about their abnormality detection processes and abnormality detection where they had no awareness of the underlying processes. Importantly, abnormality detection processes remained effective even when participants reported they were guessing, and these processes generalized to novel histopathology images.

**Fig. 5:**
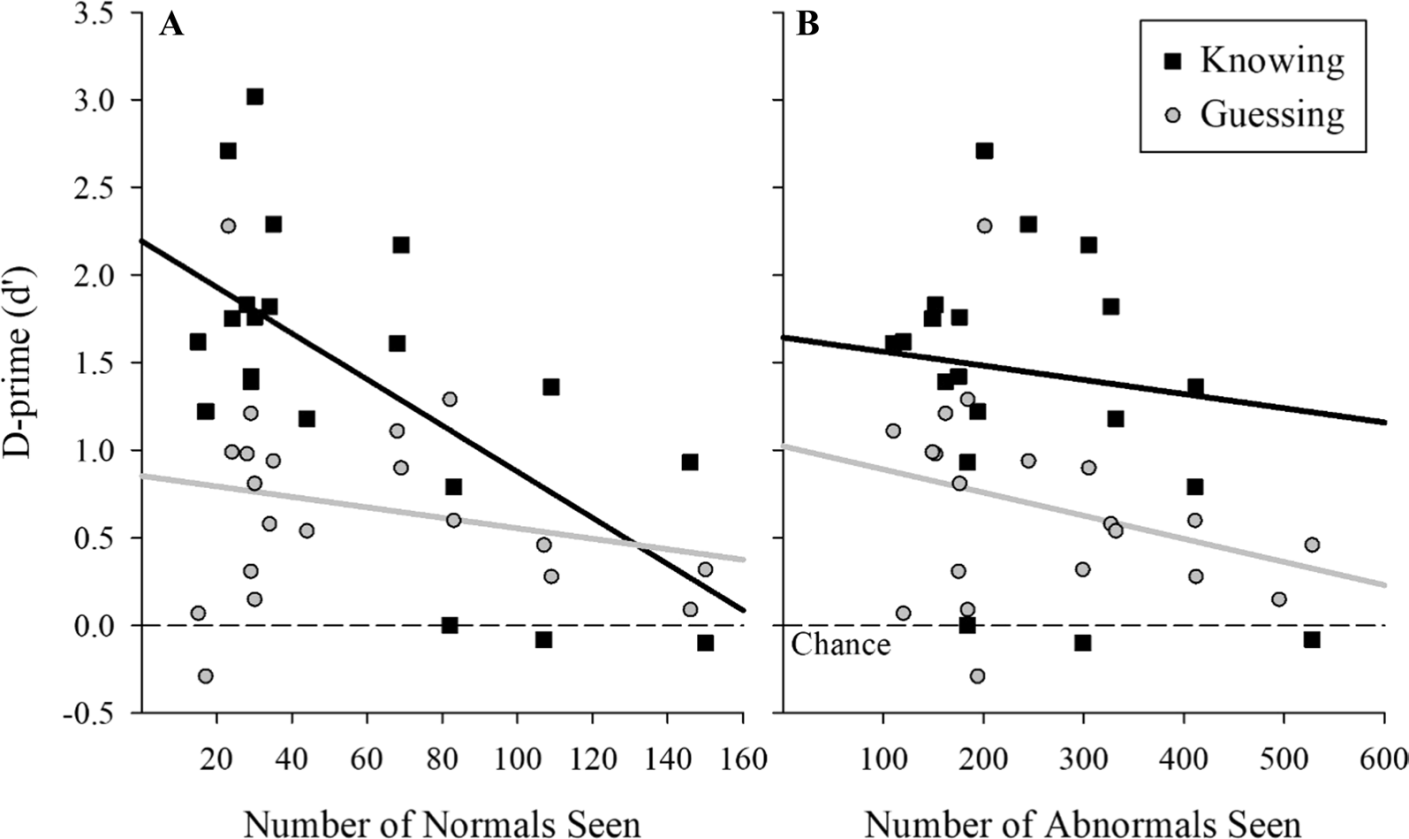
**a** and **b** Relationship between participants’ gist abnormality detection with the number of normal (left) and abnormal (right) images seen during learning, computed separately for self-reported guessing versus knowing. Black squares identify participants’ responses when they reported knowing, and the solid black line identifies the best-fitting line to these data. Gray circles and the gray line correspond to trials where the participants reported guessing. The more rapid the perceptual learning of the normal images (as indexed by less normal images seen before reaching learning criterion), the better the gist abnormality performance when participants reported knowing. No such relationship existed when participants reported guessing suggesting two separate gist processes

To determine which learning factors underlie this abnormality detection ability in novices, we conducted a regression on their abnormality detection performance with the factors from their perceptual training. When participants claimed “knowing,” the variation in abnormality detection abilities was partially explained by the number of normal images seen during training (Fig. 5a; dark lines; *b* = − 0.01, *β* = − 0.66; *t*(18) = 3.7, *p* = 0.001, *R*^2^ = 0.44), demonstrating that faster learning of the normal images produced better abnormality detection. Post hoc analyses demonstrated that familiarity did not seem to alter this relationship, based on separate stepwise regression for familiar and novel images. In both cases, the number of normal images seen during training was the only predictor included in the regression model and both indicated a negative relationship (familiar, *b* = − 0.026, *t*(14) = 8.3, *p* < 0.001; novel, *b* = − 0.012, *t*(17) = 3.8, *p* = 0.001; for these analyses, only data with at least 5 observations contributing to hit rate and false alarm rate were considered). Note, as Fig. 5b shows, there was no relationship between the number of abnormal images seen during training or how fast participants learned to detect the abnormality during training and their abnormality detection performance. These analyses suggest that when participants claimed to use a decision process that resulted in “knowing,” participants who learned to identify normal images more quickly (i.e., required fewer trials to learn which images were “normal”) performed better than those who required more training to reach criterion on learning the normal.

The analysis of “guessing” responses (see Fig. 5a, light lines) suggests an additional and alternative process contributing to participants’ performance when “knowing” is absent. The stepwise regression analysis of abnormality detection indicated no relationship with training when the participants reported guessing (correlation *r*s < 0.31, *p*s > 0.193). Because this null result may suggest an important difference in the processes generating “knowing” versus “guessing” judgments, we examined this in a Bayesian framework to identify the degree of support for the null hypothesis. This analysis showed substantial evidence (BF = 0.26, uninformative reference prior) in support of the null hypothesis when regressing these detection measures against the number of normal slides seen during training. Thus, while the normal template seems important for conscious awareness of abnormality detection, the speed of perceptual training of the normal histology image was unrelated to non-conscious abnormality detection, suggesting two distinct and separable abnormality detection processes with one explicit and one implicit.

### Abnormality categorization

This analysis, shown in the right columns of Fig. 4a, revealed clear evidence of abnormality categorization (*d*′ = 0.41, SE = 0.07; *t*(18) = 5.7, *p* < 0.001, *d* = 1.3), with abnormality categorization above chance levels of 0 (acute inflammation *d*′ = 0.69, SE = 0.15; chronic inflammation *d*′ = 0.29, SE = 0.08; cell and tissue injury/repair d' = 0.36, SE = 0.11; neoplasia *d' = 0.30, SE = 0.08*; with *t*s(18) > 3.2, *p*s < 0.005, *d*s > 0.7). A one-way repeated measures ANOVA suggested that the different categories supported different levels of abnormality categorization, *F*(3,54) = 3.8, *p* = 0.016, *η*^2^_p_ = 0.173, with uncorrected post hoc tests identifying acute inflammation as supporting superior recognition (*p*s < 0.028) and no differences among the remaining categories (*p*s > 0.608).

#### Self-reported process

Further analyzing the separate abnormality categorizations with the participants’ responses of “knowing” and “guessing” was difficult because of the low counts. Using a threshold of at least 10 items to assess hit rate and false alarm rate, four participants’ “guess” responses would be fully excluded and eight participants’ “know” responses would be fully excluded, totaling eight participants excluded on a pairwise analysis. (Requiring all four categories be present would increase this to 18 of the 20 participants being excluded.) Considering only accuracy provided more data to consider. Abnormality categorization performance could only be considered where the initial abnormal stimulus was correctly identified as containing an abnormality. Again, to ensure stability, accuracy estimates with fewer than 10 observations were excluded from the analyses.

Examining the accuracy of abnormality categorization on correctly identified abnormal stimuli revealed significantly above chance performance (*M* = 35.2%, SE = 1.9%; one-sample test comparing to chance = 25%, *t*(19) = 5.4, *p* < 0.001, *d* = 1.2). This accuracy remained significantly above chance even when participants reported guessing at the categorization (*M* = 34.1%, SE = 2.0%; *t*(17) = 4.6, *p* < 0.001, *d* = 1.1) and did not differ from when participants reported knowing the answer (*M* = 38.0%, SE = 2.8%; paired *t*(15) = 1.2, *p* = 0.256; BF = 0.36, using diffuse prior). Again, familiarity with the stimuli did not appear to affect performance (*t*(17) = 0.1, *p* = 0.945, BF = 0.18, using diffuse prior)—participants were significantly above chance when identifying the abnormality category in familiar histology images seen during training (*M* = 35.9%, SE = 2.1%, *t*(18) = 5.2, *p* < 0.001, *d* = 1.2) and, more critically, novel histology images were also categorized significantly greater than chance (*M* = 35.5%, SE = 2.8%, *t*(17) = 3.7, *p* < 0.003, *d* = 0.87).

Next, we considered how the participants’ accuracy in their abnormal categorization related to their performance during learning (note, dividing the data by familiar versus novel would include only six participants with complete data to consider, so that analysis is omitted). Here, when participants reported guessing, our learning metrics showed no predictive value (*r*s < 0.344, *p*s > 0.162, BFs < 0.47, using uniform prior), but when they reported knowing, the story was more complex. The final model in this latter case included a negative relationship with the number of normal images seen during training (*b* = − 0.001, *β* = − 0.50; *t*(14) = 2.8, *p* = 0.015) and a positive relationship with the time spent learning (in seconds, *b* = 7.2× 10^−5^, *β* = 0.69, *t*(14) = 3.9, *p* = 0.002; overall model *R*^2^ = 0.58). Here again, better performance was found with faster learning of the normal images, and the categorization seemed to be improved when participants spent more time during the learning. Crucially, categorization performance was seemingly uncorrelated with the number of abnormal stimuli seen (*r* = 0.19, *p* = 0.466, BF = 0.24, using uniform prior), suggesting that perhaps the participants’ time spent processing the stimuli during the PALM feedback improved their categorization performance. As before, these analyses suggest distinct and separable abnormality categorization processes.

Finally, we separated the number of times participants correctly or incorrectly categorized the abnormality based on their self-reported “guessing” and “knowing” for both the abnormality detection and category judgment (Table 1 presents these numbers as relative frequencies). Table1 suggests that the “knowing” and “guessing” for the two decisions were not always in agreement (*χ*^2^(1) = 409, *p* < 0.001). Though the majority (69.4%) of self-reported “guessing” and “knowing” responses were the same in the two decisions, a considerable number diverged. The majority of trials (29%) were “guessing” with correct abnormality detection and “guessing” with incorrect categorization. There were a fair proportion of trials (10%) in which the participants got both decisions correct and reported “knowing” during both decisions. As we shall cover in the discussion, we believe these different types of responses are suggestive of multiple processes occurring some of which have been defined in the literature as a global implicit signal (“guess”) without localized information about the abnormality (Evans et al., 2013, 2016, 2019), some of which are a global signal that leads to a conscious detection (“know”) of local information of the abnormality (Kundel & Nodine, 1975; Kundel et al., 2008) and some of which are neither.

**Table 1:**
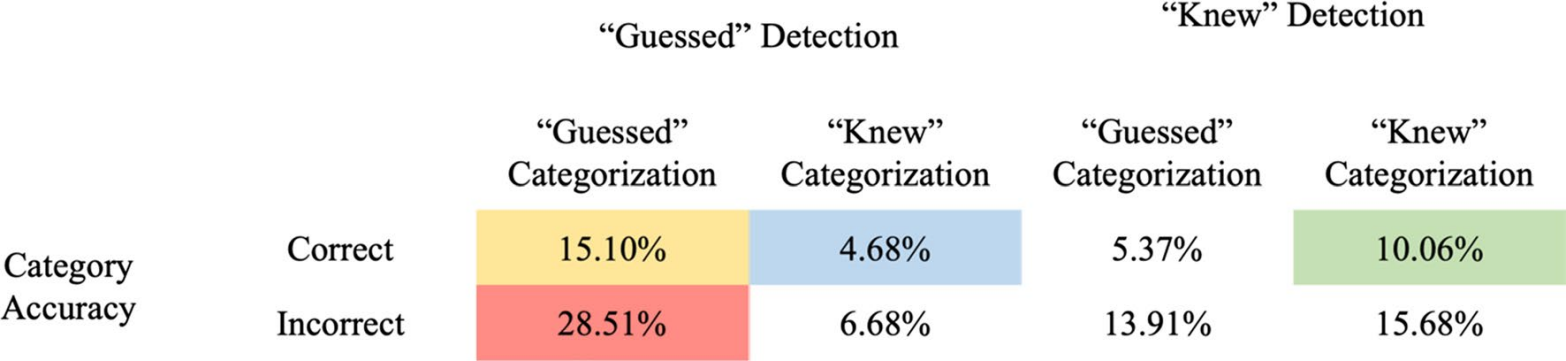
Frequencies of category decisions following correct abnormality detection

## Discussion

The aim of this study was to determine if global implicit abnormality processing occurs in naïve (non-medically trained) participants who received a brief perceptual training and to tease apart the nature of that signal. Perceptual learning of histology images in medical students has previously produced marked improvement in histopathology performance (Krasne et al., 2013). We found that naïve participants who are briefly perceptually trained also show good learning of histopathology. The data were telling in that perceptually trained naïve participants also showed significant abnormality detection for histology images. Even with perceptual training lasting less than 1 h, these naïve participants were able to detect a skin histopathology well above chance with a brief 500 ms presentation and visual masking. These results in naïve participants mirror a study by Brunye et al. (2021) who showed that experts (resident and attending pathologists) showed abnormality detection with a 500 ms presentation of a histopathology, suggesting our perceptually-trained naïve participants were performing comparably. Of course, our participants did not have years of medical training, nor did they have many thousands of exposures to histopathology images, suggesting that the development of abnormality detection is rather rapid when perceptually trained and does not require extensive exposure.

Further, the abnormality detection in our naïve participants generalized to novel histopathology images. These data indicate that our participants had developed a general abnormality detection process and were not using solely memory processes or matching of specific images. The generalization of processes learned through perceptual training has previously been demonstrated for abnormality detection with long exposure durations (Johnston et al., 2020; Xu et al., 2016), but this is the first example of a generalizable rapidly developed abnormality detection process.

Another aim of this study was to parse this signal and understand what drives its development. Here the data were telling. When making their abnormality responses, participants also reported whether they knew, or if they were guessing. In both cases, naïve participants demonstrated significant abnormality detection above chance levels. To be explicit, even when naïve participants reported they were just guessing, they still showed significant abnormality detection. These data are in line with the notion of an implicit global signal (“gist”) as defined by Evans and colleagues (Evans et al., 2013, 2016, 2019) which suggest an implicit global signal without awareness of the exact location or type of abnormality. Further, regression analyses demonstrated that there were two distinct processes underlying abnormality detection. When participants reported guessing, no factors related to their perceptual learning were significant predictors of the amount of abnormality detection participants showed. However, when naïve participants reported knowing, their abnormality detection was directly negatively related to the number of normal images they had seen during training (see Fig. 5a, dark line), with fewer normal images seen during training producing larger *d’* scores for abnormality detection. Recall that in the adaptive perceptual learning, participants retired a category when they recorded three consecutive correct answers for that category. Hence, those naïve participants who had seen fewer normal images during perceptual learning were also the participants who had learned the normal histology images fastest. Our data are clear that faster learning of normal histology images produced larger *d*′ scores and hence demonstrate more abnormality detection. These data agree with the prediction of Kundel and Nodine (1975) and Kundel et al. (2007, 2008): Better abnormality detection arises from a better normal template. Kundel and colleagues have argued that the violation of the normal template creates a global signal that directs selective attention to the location of the abnormality.

However, these data also show that there is more than one process that produces abnormality detection, as trials where participants were guessing showed no such relationship with the normal template. In our view, more than one signal is created in the visual system, with one global signal supporting a general abnormality process (trials where participants guessed and had no local information about the type of abnormality). These trials are shown in pink in Table 1 where participants reported “guessing” on the abnormality detection but get it correct, and then get the category wrong while reporting guessing for the category. These data are evidence of an implicit global signal without local information (Evans et al., 2013, 2016, 2019). But an additional signal giving rise to identification of the locations of abnormalities is also seen. As shown in Table 1 (in blue and green), participants correctly identify the category of the abnormality and report “knowing.” The local information needed to identify the category could arise from two potential mechanisms. First, as suggested by Kundel and Nodine (1975) and Kundel et al. (2007, 2008), the global “gist” signal gives the location information of where the abnormality lies. As the global signal arises as a comparison of the normal template against the current image and that comparison violation has a specific location. Alternatively, after a global signal, participants might be randomly foveating different regions of the histology image and hence find the location where the abnormality lies. Because the image is up for only 500 ms, only one or two eye movements are likely (Brunye et al., 2021). However, participants report “knowing” that it was an abnormality and “knowing” during categorization is clear evidence for additional processes besides the global implicit signal as proposed by Evans et al. (2013, 2016, 2019). Further evidence for multiple processes comes also from the results of the categorization.

If the global signal only produced a general abnormality signal, then participants would be at chance for discriminating the type of histopathology that was present. However, our naïve but perceptually trained participants showed clear evidence of category identification, with values that were above chance even with a single brief 500 ms presentation of a histology image that was then visually masked. Given that the category identification came after the abnormality judgment and the reporting of how they made this abnormality judgment, we suspect that their performance would have been even higher if the category identification were made immediately after the 500 ms presentation. It is also likely that given the categorization occurs after the stimulus has been masked and after participants have made the detection judgment and reported whether they were “guessing” or “knowing,” the information may be decaying and impeding their ability both to make the categorization and to use self-report accurately. Nevertheless, perceptually trained naïve participants can still discriminate among four histopathology categories suggests that at least one of the signals must contain some local information about the abnormality that allows discrimination between the four pathologies. As shown in Fig. 1, the abnormality categorization requires local information about the region of the histology image where the abnormality occurs. Note, however that these data also suggests two separate processes. When we decomposed the abnormality detection into trials where participants reported “knowing” versus “guessing” the answer, we also get two distinct processes. For abnormality categorization where participants reported they were merely “guessing,” naïve participants were significantly better than chance at discriminating the specific histopathology category. However, these trials were unrelated to the factors associated with their perceptual learning. In contrast, when naïve participants reported “knowing” during abnormality categorization, they were again significantly better than chance and their performance was again related to how rapidly they learned the normal template (Kundel & Nodine, 1975; Kundel et al., 2007, 2008). Carrigan et al. (2018) have also demonstrated that the global signal contain location information. The current data are clear that multiple processes are developed rapidly after perceptual training, and these different signals are dissociable and support different processes. It is likely that a global signal is present and a more specific signal that also contains location information is derived from every medical image. Note we also have evidence of an implicit global and local signal. As Table 1 also shows (in yellow), there are a number of trials in which participants report “guessing” for both the abnormality and the category and get both of them right. These data are supportive of an implicit signal at both the global and local scale that can support both abnormality detection and abnormality categorization in the absence of explicit information. We must be vigilant of over-interpreting some of our results as some of these difference relies, in part, on the participants self-report of whether they knew or guessed. However, participants were significantly better when they reported “knew” than “guessed,” suggesting some accuracy in these judgments. The current data are clear evidence of multiple processes arising from perceptual training in novices which support abnormality detection and abnormality categorization.

It is our contention that experts may also derive multiple signals during the many learning trials associated with the tens of thousands of images that they are exposed to throughout their training and careers. It is important to note that in the real world, medical experts may rely far less on these global signals than our participants. However, there is speculative and anecdotal evidence that these processes play a role. While not technically limited to 500 ms for each image, experts are under a lot of time pressure to read quickly. For example, a typical day of radiology reading might be 30 CTs acquired at 1 mm per day, translating to ~ 10,320 images read over a 5-h period, leaving little time for each slice. Additionally, one of the authors (MR) is a practicing radiologists and reports that when viewing cases, he sometimes gets a nagging feeling that something is wrong and goes back and finds an abnormality.

The debate over the nature of the gist signal of a global implicit abnormality signal (Evans et al., 2013, 2016, 2019) versus a global signal that is compared to a normal template and provides specific location information for attention (Kundel & Nodine, 1975; Kundel et al., 2007, 2008) may be so contentious because more than one signal is evident. In the current data, dissociable signals are seen for abnormality detection and for abnormality categorization. The ability of our naïve participants to correctly identify the category of the abnormality and the dissociation in abnormality detection between guess and know trials supports the idea of multiple processes (both global and local and both implicit and explicit, see Table 1). The finding that our participants are able to correctly classify an image as abnormal even when they report that they are just guessing also supports a general global process. In short, our data support both arguments but only because multiple signals are evidenced. That multiple signals are produced after a brief perceptual training speaks to the power and utility of perceptual learning and the remarkable adaptability of the human visual system.

## Conclusion

We explored the ability of naïve participants to rapidly acquire visual expertise in medical images after a brief perceptual and adaptive learning procedure. We find that perceptual learning produces significant changes in the human visual system for medical images and gives rise to multiple, distinct global signals that can be used to identify whether an abnormality is present and what type of abnormality is present. This rapid acquisition of multiple global and local perceptual processes in naïve participants has critical implications for how radiologists and pathologists are trained. To date, the trainings of radiologists and pathologists are partially dependent on exposure to whatever abnormalities are present during their residency or whatever current elective they are pursuing. The present data suggest that a brief concentrated perceptual training can produce multiple dissociable visual processes that support abnormality detection and abnormality categorization. And at least some of these processes are directly related to how well the participants learn a normal template. In line with Kundel and Nodine (1975) and Kundel et al. (2007, 2008), we suggest that a directed learning of a normal template prior to the learning of abnormalities may produce better abnormality detection and identification in radiologists and pathologists. We also suggest that multiple visual processes are developed during perceptual learning, some of which support conscious recognition (knowing) and some of which form non-conscious detection (guessing), and these processes are a crucial part of abnormality detection. Creating methodologies to tap into all of these processes in medical visual search might change clinical practice, reduce misses, improve early detection, and increase survival rates.
